# Importance of CD200 expression by tumor or host cells to regulation of immunotherapy in a mouse breast cancer model

**DOI:** 10.1371/journal.pone.0171586

**Published:** 2017-02-24

**Authors:** Anna Curry, Ismat Khatri, Olha Kos, Fang Zhu, Reginald Gorczynski

**Affiliations:** 1 University Health Network, Department of Surgery, Transplant Research Division, Toronto, Canada; 2 University of Toronto, Department of Immunology, Toronto, Canada; Mie University Graduate School of Medicine, JAPAN

## Abstract

Cell-surface CD200 expression by mouse EMT6 breast tumor cells increased primary tumor growth and metastasis to the draining lymph nodes (DLN) in normal (WT) BALB/c female recipients, while lack of CD200R1 expression in a CD200R1^-/-^ host negated this effect. Silencing CD200 expression in EMT6^siCD200^ tumor cells also reduced their ability to grow and metastasize in WT animals. The cellular mechanisms responsible for these effects have not been studied in detail. We report characterization of tumor infiltrating (TILs) and draining lymph node (DLN) cells in WT and CD200^-/-^ BALB/c mice, receiving WT tumor cells, or EMT6 lacking CD200 expression (EMT6^siCD200^ cells). Our data show an important correlation with augmented CD8^+^ cytotoxic T cells and resistance to tumor growth in mice lacking exposure (on either host cells or tumor) to the immunoregulatory molecule CD200. Confirmation of the importance of such CD8^+^ cells came from monitoring tumor growth and characterization of the TILs and DLN cells in WT mice challenged with EMT6 and EMT6^siCD200^ tumors and treated with CD8 and CD4 depleting antibodies. Finally, we have assessed the mechanisms(s) whereby addition of metformin as an augmenting chemotherapeutic agent in CD200^-/-^ animals given EMT6 tumors and treated with a previously established immunotherapy regime can increase host resistance. Our data support the hypothesis that increased autophagy in the presence of metformin increases CD8^+^ responses and tumor resistance, an effect attenuated by the autophagy inhibitor verteporfin.

## Introduction

Mouse models of breast cancer have provided insights into the mechanisms of immune responses to tumor cells, with the expectation that these findings may translate into more effective cancer immunotherapy in humans. EMT6 is a transplantable breast cancer cell line considered to be a less aggressive type of breast cancer compared with other cell lines, such as 4THM, which may be a closer model of rare human inflammatory breast cancer [1]. We have previously reported that cell-surface CD200 expression by mouse EMT6 breast tumor cells increased primary tumor growth and metastasis to the draining lymph nodes (DLN) in both WT and CD200^tg^ BALB/c female recipients [2]. Lack of CD200R1 expression in a CD200R1^-/-^ host negated this effect [3]. Furthermore, silencing CD200 expression in EMT6^siCD200^ tumor cells reduced their ability to grow and metastasize in WT animals [3]. These data were consistent with the hypothesis that CD200 expression, through engagement of CD200R1, leads to attenuation of a protective anti-tumor response and was important for controlling metastasis, though more details on the mechanism(s) contributing to these effects remained unexplored, particularly with respect to the importance of host vs tumor CD200 expression in regulation of host tumor resistance.

We subsequently extended these earlier findings to a model in which anti-EMT6 tumor immunity was explored in CD200^-/-^ mice and CD200R^-/-^ receiving immunotherapy (with irradiated tumor cells and CpG as adjuvant) following surgical resection of tumor [4]. While complete cure was achieved in CD200R^-/-^ mice with this regimen, in CD200^-/-^ mice the same protocol was able to decrease EMT6 metastasis, but was insufficient for generating a long-lasting anti-tumor immune response [5]. Treatment of CD200^-/-^ tumor-bearing mice by immunotherapy in combination with conventional cytotoxic chemotherapy cured primary tumors, but produced no long-lasting immunity [5]. Again we wondered whether this reflected a greater importance to tumor (vs host) CD200 expression in regulation of breast cancer growth in vivo.

Recent studies using metformin as an augmenting chemotherapeutic agent in breast cancer have produced some quite novel findings. Metformin inhibited the growth of a subpopulation of breast cancer initiating cells in culture and reduced their ability to form tumors in mice [6]. Metformin has been reported to inhibit angiogenesis and metastatic growth of breast cancer by targeting both the tumor cells and the white adipose tissue endothelial progenitor cells in the tumor microenvironment [7]. When combined with trastuzumab, metformin reduced the cancer-initiating cell population in HER2/neu-amplified breast cancer cells [8]. Metformin has also been shown to reduce the growth of a variety of tumor xenografts in mice, including those established from breast and prostate cancer cells [9,10], and suppress the development of breast, colon, and other tumors in transgenic mice [11,12].

Since CD200 can be expressed by both host cells and breast tumor cells *in vivo*, we hypothesized that the location of expression of CD200 and/or CD200R contributes importantly the mechanism(s) triggered which might lead to host tumor resistance, and help explain some of our earlier findings cited above. Moreover, we speculated that use of a chemotherapeutic agent not itself acting primarily as a cytotoxic drug (e.g. metformin), might lend itself to further manipulation of host resistance which would manifest in prolonged protection even in CD200^-/-^ mice. The current study was designed with the following goals in mind:

1. to characterize tumor infiltrating lymphocytes (TILs) and draining lymph node (DLN) cells in WT and CD200^-/-^ BALB/c mice, receiving WT tumor cells, or EMT6 lacking CD200 expression (EMT6^siCD200^ cells).
2. to explore a protective role for CD8^+^T cells in the anti-EMT6 tumor immune response, and assess a contrary role for CD4^+^Tregs, by characterizing the TILs and DLN cells in WT mice challenged with EMT6 and EMT6^siCD200^ tumors and treated with CD8 and CD4 depleting antibodies.
3. to explore whether addition of metformin, a novel agent thought to promote autophagy, which itself has been linked with CD8^+^T cell survival, can increase host resistance in CD200^-/-^ animals given EMT6 tumors and treated with a previously established immunotherapy regime.

## Materials and methods

### Mice

Female WT BALB/c stock mice were purchased from Jackson Laboratories (Bar Harbor, Maine). CD200^-/-^ (BL/6 background) mice were derived commercially by Caliper Life Science, with deletion of exons 2–4 encoding murine CD200 [5]. CD200^-/-^ BALB/c mice were derived from founder stock (on BL/6 background) and backcrossed through ten generations with BALB/c mice obtained from Jax Labs before intercrossing for use in subsequent studies [5]. All mice were housed 5/cage and allowed food and water *ad libitum* in an accredited facility at the University Health Network (UHN). Female mice were used at 8–12 weeks of age. All animal experimentation was performed following guidelines, and approval, of a CCAC accredited animal care committee at University Health Network (UHN), (approved protocol No. AUP1.15). Humane endpoints were used in all studies, with mice monitored daily. Animals were euthanized (overdose with pentobarbital) when they were exhibiting signs of distress (weight loss≥25%; hunched posture; diarrhea; loss of active movements), or if tumor size exceeded 1.5cm^3^, and at the pre-determined endpoint of the studies. No unanticipated mortality was seen in any of the studies described and all animals survived to the pre-set endpoint in each study.

### Tumor cells and growth of EMT6 in mice

The BALB/c-derived EMT6 breast tumor cell line was obtained from ATCC and passaged *in vitro* in α-minimal essential medium (α-MEM) with 10% fetal calf serum (αF10), supplemented with penicillin (10 units/ml) and streptomycin (10μg/ml).

EMT6^siCD200^ cells were derived as described previously [3] and passaged *in vitro* in αF10, supplemented with penicillin (10 units/ml) and streptomycin (10μg/ml), as well as puromycin (1μg/ml) for selection and maintenance of silenced cells. Cells were incubated in a humidified CO_2_ incubator at 37°C.

5 × 10^5^ EMT6 tumor cells in 150 μl of PBS were injected into the mammary fat pad of recipient mice. Seventeen to 22 days post tumor cell injection, mice were euthanized, tumors were excised, and draining lymph nodes and contralateral lymph nodes (CLN) were harvested. Single-cell suspensions from the lymph nodes were prepared by passing the tissues through 70 μm filters and suspending the cells in PBS. Tumor cells were digested with a mixture of collagenase and trypsin for 30 min at 37°C in a bottle with a magnetic stirrer. Tumors cells were then centrifuged over mouse lymphopaque (Cedarlane Labs, Hornby, Ontario, Canada) and suspended in PBS without calcium and magnesium.

Micro-metastases to draining lymph nodes (DLNs) in tumor bearing mice were measured at 14d using cells harvested from individual mice and cloned at limiting dilution in microtiter plates for 21days as described in earlier reports [2–4]. Except where specified macro-metastases to lung/liver were enumerated visually on tissues harvested and fixed in Bouin’s solution.

### Tumor resection and immunotherapy of treated mice

EMT6 and EMT6^siCD200^ tumors were surgically resected from WT BALB/c and CD200^-/-^ mice 12–15 days post tumor cell inoculation [4]. Mice receiving immunotherapy were treated by intraperitoneal (ip) immunization with 2x10^6^ EMT6 tumor cells (irradiated with 2500 rad) mixed with 100 μg CpG ODN in 100 μl PBS and emulsified with an equal volume of Incomplete Freund’s adjuvant, 1–2 days after surgery [4]. In some studies, metformin, an agent reported to be able to attenuate breast cancer cell growth, was also administered daily (60 mg/kg ip) for an additional 6 weeks. In further studies Verteporfin, an autophagy inhibitor, was used (4.5 mg/kg ip, for a total of 3 doses at 60hr intervals), in mice treated with/without metformin. In these investigations all mice were kept in a darkened environment for 24 hrs post injections.

Where Western blots were used to detect evidence for autophagy, a rabbit antibody to LC3 was used (BioLegend, USA). Increased LC3 conversion (LC3-I to LC3-II) is a marker of augmented autophagy, with the amount of LC3-II correlated with the number of auto phagosomes. Note that despite the fact that LC3-II has a higher molecular weight than LC3-I it is known to migrate more rapidly in SDS-PAGE compared with LC3-I, likely because of the greater hydrophobicity associated with the phosphatidylethanolamine group.

### Immunostaining and flow cytometry

Single-cell suspensions from DLN, CLN, and tumors were washed twice with 1 mL FACS buffer (PBS, 5% FBS, 5mM EDTA) and incubated with cell-surface antibodies (or isotype controls) purchased from Biolegend (see below) at concentrations recommended by the supplier for 20 min at 4°C in the dark. Cells were washed again and fixed with 1% paraformaldehyde (PFA) for 30 min. Samples were assessed by a LSR flow cytometer and the data were analyzed using FlowJo software. Analysis gates were set with the aid of “fluorescence minus one” isotype controls. Forward scatter pulse height and side scatter analyses were performed to exclude cell clusters.

### Antibodies used for flow cytometry

Anti-CD45, anti-CD3, anti-CD4, anti-CD8, anti-CD11b, anti-GR-1, anti-F4/80, and anti-CD200 antibodies conjugated to PE, FITC, APC, Pe-Cy7, or APC-Cy7 fluorochromes were purchased from Biolegend and used at concentrations determined recommended by the supplier for immunostaining and flow cytometric analysis of DLN, CLN, and tumor cells.

Anti-CD4 (L3/T4, clone YTS) mAb ascites purchased from Cedarlane, and anti-CD8a monoclonal antibody (clone 53–6.7) purchased from Biolegend, were used for depleting CD4^+^ or CD8^+^ T cells respectively in tumor-bearing mice using 50μg antibody/mouse iv at 96hr intervals for 3 injections, beginning 6 days post tumor inoculation).

Intracellular staining for IFN-γ and FOXP3 was performed using the BD Biosciences Cytofix/Cytoperm Kit (Cat # 555028) according to the manufacturer’s instructions after incubating DLN cells with BD GolgiPlug for 8 hrs (Cat#555029).

### Proteomics and ELISA analysis

EMT6 and EMT6^siCD200^ tumor bearer lysates were assayed for the expression of 40 proteins associated with inflammation using Proteome Profiler Mouse Cytokine Array Panel A purchased from R&D Systems (catalog #ARY006), following the manufacturer’s instructions.

Supernatants of EMT6 and EMT6^siCD200^ tumors collected after 24 hrs in αF10 culture were assayed for the expression of 12 mouse inflammatory chemokines and cytokines using a Multi-Analyte ELISArray Kit purchased from Qiagen. These same supernatants were also assayed for GM-CSF and IL-1β concentration using ELISA kits purchased from Biolegend. A standard curve was obtained in each assay to quantify cytokines/chemokines present in the supernatants.

## Results

### Characterization of BALB/c CD200^-/-^ mice, and of tumor growth in those mice

The gross cellular phenotype of male and female CD200^-/-^ BALB/c mice ~12 weeks old was assessed by flow cytometry of immune cell populations and ELISA of sCD200 in peripheral blood. In comparison to WT mice, with ~5–8 ng/ml of circulating sCD200 in their peripheral blood, CD200^-/-^ mice had undetectable levels of sCD200 (S1 Fig). Flow cytometric analyses of spleen, thymus, as well as axillary and mesenteric lymph nodes confirmed the lack of cell-surface CD200 expression in CD200^-/-^ mouse organs.

**Fig 1:**
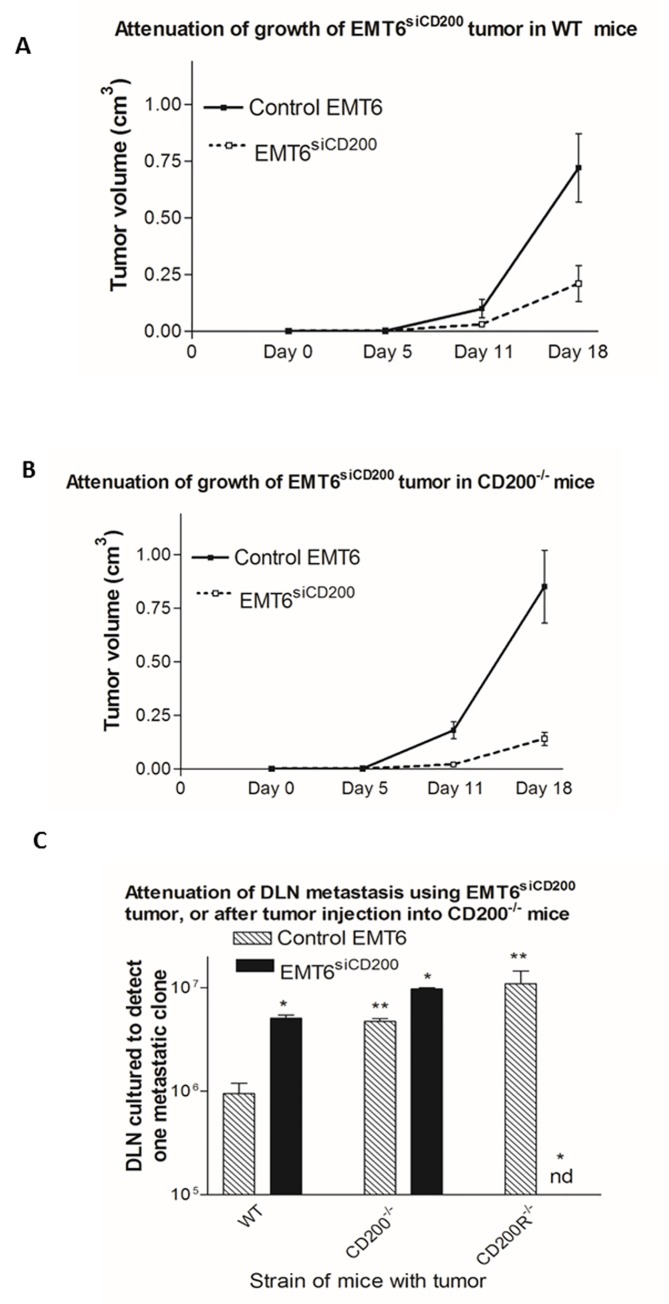
Comparison of growth of EMT6 and EMT6^siCD200^ tumors in WT (A) and CD200^-/-^ (B) female mice. All values show (mean±SD) for 4 mice/group-data are representative of 4 independent studies. Data in panel C show DLN metastases (as number of DLN cells cultured/tumor clone detected) determined in limiting dilution studies in WT, CD200^-/-^ or CD200R^-/-^ mice (using both control and EMT6^siCD200^ tumors). * indicates p<0.05 compared with control EMT6 tumor cells; nd = no metastases detected. ** indicates p<0.05 compared with control EMT6 tumor in WT mice.

When tumor growth was compared in WT and CD200^-/-^ mice, the rate of growth of control EMT6 and EMT6^siCD200^ tumor cells was found to be similar in each strain, with EMT6^siCD200^ tumors consistently smaller in both WT and CD200^-/-^ hosts at the time of sacrifice (Fig 1). Thus control EMT6 tumors growing in immunocompetent WT female BALB/c mice reach upwards of 0.7cm^3^ by day 18 post tumor cell injection (Fig 1A), with similar growth in CD200^-/-^ mice (Fig 1B). In contrast, EMT6^siCD200^ tumors at that time-point were <0.25cm^3^ in both WT and CD200^-/-^ mice, implying an important role for tumor CD200 expression in regulation of tumor growth in both strains. We did note, as reported elsewhere [4,5] a difference in the ability of EMT6 and EMT6^siCD200^ tumors to metastasize to DLN in CD200^-/-^ vs WT mice (Fig 1C), with even control EMT6 metastasizing less in CD200^-/-^ mice, indicating an importance for both host and tumor CD200 expression in this phenomenon.

### Analysis of cell populations harvested from tumors in WT and CD200^-/-^ BALB/c female mice

**Fig 2:**
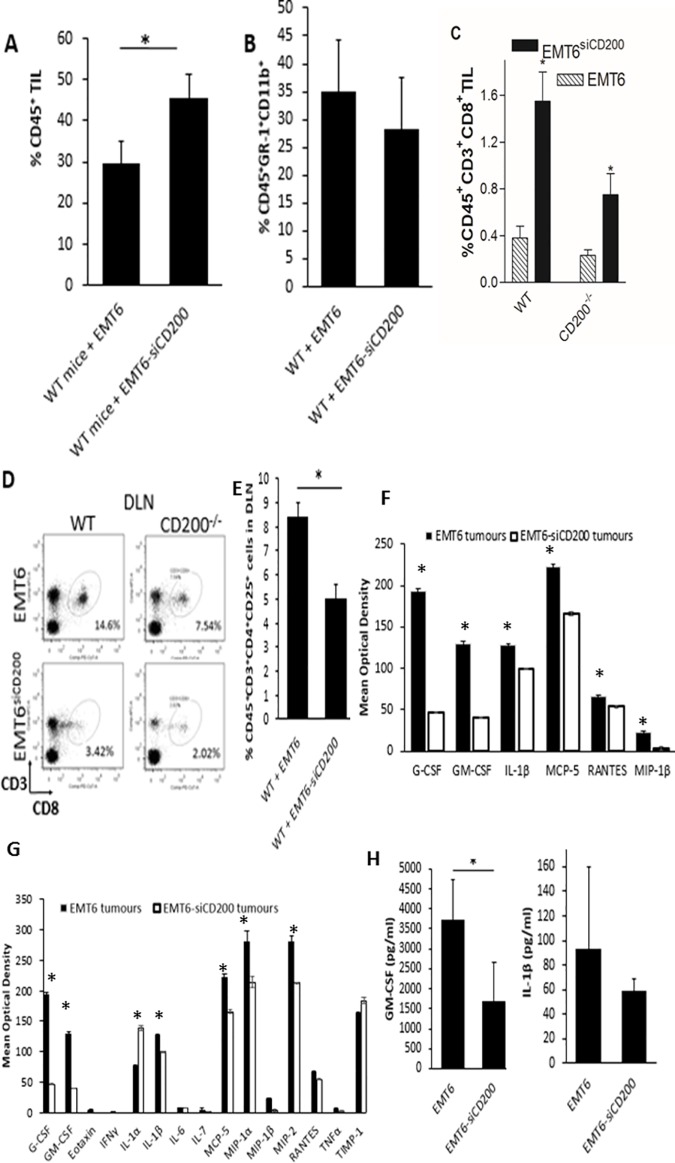
EMT6 and EMT6^siD200^ tumor microenvironments in WT and CD200^-/-^ female mice. A) EMT6 and EMT6^siCD200^ tumors were harvested from WT mice at day 20 post tumor cell injection. Tumors were digested, stained for the CD45 cell-surface molecule, and analyzed by flow cytometry. Data show mean (±SD) of 4 mice/group. *p<0.05. B) and C). EMT6 and EMT6^siCD200^ tumors grown in WT and CD200^-/-^ mice were digested and stained for CD45^+^GR-1^+^CD11b^+^ cells (b), and CD45^+^CD3^+^CD8^+^ (c) cells. Panels show (mean±SD) from 4 mice/group after gating on CD45^+^ cells. *p<0.05, **p<0.01. D) and E). DLN from WT and CD200^-/-^ mice with EMT6 and EMT6^siCD200^ tumors were digested and stained for CD45, CD3, CD4^+^, CD25^+^ and CD8 cell-surface markers. Panels in D) show representative dot plots from 4 mice/group after gating on CD45^+^ cells. Data in E) shows % CD4^+^CD25^+^ cells in DLN gated on CD45^+^CD3^+^ cells. >25% stained by intracellular staining for Foxp3^+^ (not shown). F) Relative levels of G-CSF, GM-CSF, IL-1β, MCP-5, MIP-1β, and RANTES expressed in EMT6 and EMT6^siCD200^ tumors grown in WT mice were assessed using a proteomics array. Data show mean (**±**SD) optical density (OD) from 3 independent tumor samples of each type. * indicates p<0.05 compared with EMT6^siCD200^ tumors. G) Equivalent proteomics array (to that shown in panel F) performed using aliquots of 24hr supernatants from tumor cells shown in panel F). Data show mean (**±**SD) of 4 tumors/group. * indicates p<0.05 compared with EMT6^siCD200^ tumors. H) Levels of GM-CSF and IL-1β in 24hr supernatant (panel G) from EMT6 and EMT6^siCD200^ tumors cultured in αF10 as assessed by ELISA. Data show mean (**±**SD) of 4 tumors/group. *p<0.05.

Previous studies in both WT mice, and in CD200R^-/-^ mice, had implied a role for both GR-1^+^CD11b^+^ (myeloid-derived suppressor cells, MDSCs) and CD8^+^ T cells in host resistance to EMT6 tumor cell growth in this model [2–5]. We next examined tumor infiltrating lymphocytes (TIL) and immune cells in the DLN and contralateral lymph nodes (CLN) in tumor-bearing mice by cell-surface staining and flow cytometry. While there were fewer cells (~20% decrease-data not shown) infiltrating EMT6^siCD200^ tumors we observed a higher percentage of CD45^+^ immune cells than in control EMT6 tumors (Fig 2A). These CD45^+^ cells included macrophages (GR-1^+^F4/80^+^) and GR-1^+^CD11b^+^ cell (Fig 2B) and CD3^+^CD8^+^T cells (Fig 2C). The TIL in EMT6^siCD200^ tumors contained less MDSC (Fig 2B) with more enrichment for CD8^+^ T lymphocytes (Fig 2C) than TIL from control EMT6 tumors, in both WT and CD200^-/-^ mice. This corresponded with the lower numbers of CD8^+^ T cells found in the DLN of mice bearing EMT6^siCD200^ tumors (Fig 2D). In contrast, we detected more CD4^+^CD25^+^ T_reg_ cells in the DLN of WT mice bearing EMT6 tumors compared to EMT6^siCD200^ tumors (Fig 2E). These results are consistent with the hypothesis that adaptive immunity is enhanced in hosts carrying tumors that have reduced expression of the immunoregulatory CD200 molecule. Note however than functional activity of CD8^+^ cells in TIL and/or DLN, and of CD4^+^CD25^+^ T_reg_ cells in the DLN were not assessed in these studies (see later Figures).

The relative levels of 40 different cytokines, chemokines, and acute phase proteins present in the microenvironment of EMT6 and EMT6^siCD200^ tumors growing in WT female mice were determined using a commercial proteomics array with tumor lysates. There was a decrease in the concentration of several soluble mediators in EMT6^siCD200^ tumor lysates compared with EMT6 lysates, including G-CSF, GM-CSF, IL-1β, MCP-5, MIP-1β, and RANTES (Fig 2F). Given the correlation with the increased immune cell infiltration and decreased tumor size in EMT6^siCD200^ tumors, these data are consistent with a role for these cytokines in promoting EMT6 tumor growth in female WT BALB/c mice.

Supernatant samples from cultures of EMT6 and EMT6^siCD200^ tumors kept in αF10 medium for 24 hrs were also assayed for cytokines and chemokines detected by a commercial mouse inflammatory protein ELISA. Consistent with the results from the proteomics array, GM-CSF and IL-1β levels were decreased in supernatant samples from EMT6^siCD200^ tumors compared with control EMT6 tumors (Fig 2G). Using quantitative ELISAs of mouse IL-1β and GM-CSF, control EMT6 tumors expressed more GM-CSF than EMT6^siCD200^ tumors cultured for 24 hrs (3.74 ± 1.01 ng/ml vs. 1.7 ± 0.9 ng/ml), and showed a trend to express more IL-1β than EMT6^siCD200^ tumors (93 ± 66 pg/ml vs. 58 ± 10 pg/ml) with significant individual variability in the data collected (Fig 2H).

### Role of CD4^+^ and CD8^+^ T cells in host resistance to EMT6 or EMT6^siCD200^ tumors

Reduced expression of CD200 by EMT6 tumor cells resulted in decreased local tumor growth and metastasis tumor size (Fig 1), which we hypothesized may reflect an enhanced adaptive anti-tumor immune response to EMT6^siCD200^ cells compared with control EMT6 cells. Indeed, in studies using a combined surgery and immunotherapy regime to cure CD200R^-/-^ mice of tumors, both CD4^+^, and less importantly CD8^+^ cells, were found to be implicated in host resistance [4]. To investigate further a role for CD8^+^ and CD4^+^ T cells in mediating anti-tumor responses in WT mice, we treated EMT6 and EMT6^siCD200^ tumor bearing animals with either an anti-CD8 or anti-CD4 depleting antibody beginning at day 6 post tumor cell injection (x3 injections).

**Fig 3:**
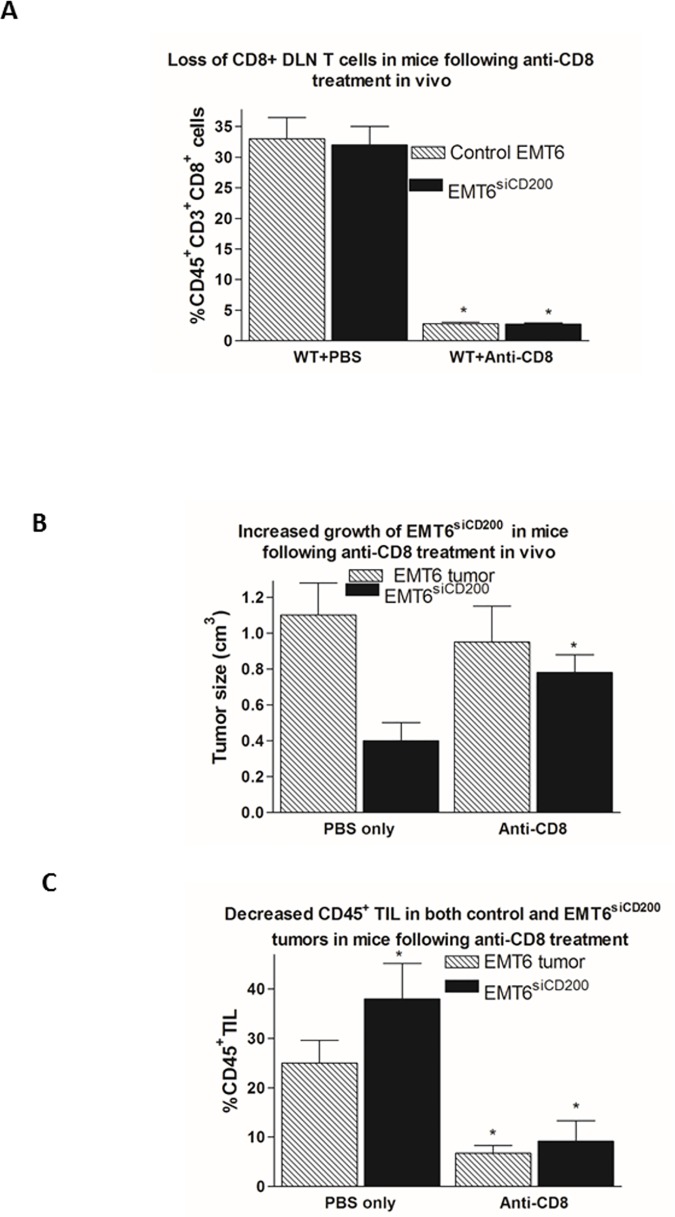
Effect of depletion of CD8^+^ T cells on WT host resistance to EMT6 and EMT6^siCD200^ tumors. A. Staining for CD45, CD3, and CD8 cell surface markers in single cell suspensions of DLN from EMT6 and EMT6^siCD200^ tumor bearing WT mice treated with anti-CD8 antibody or PBS. Data show mean (**±**SD) of 3 mice/group. * p<0.01 compared with equivalent PBS controls. B. Tumor size (day 19) in mice from panel A after treatment with anti-CD8 or PBS. Data show mean (**±**SD) of 3 mice/group. *p<0.05 compared with equivalent PBS control. C. CD45^+^ TIL in EMT6 and EMT6^siCD200^ tumors harvested from mice in panels A and B, after treatment with anti-CD8 or PBS. Tumors were digested, stained for CD45 cell-surface expression, and analyzed by flow cytometry. Data show mean (**±**SD) of 3 mice/group. *p<0.05 compared with equivalent PBS control.

DLNs of tumor bearing mice treated with anti-CD8 showed > 90% depletion of CD8^+^ T cells (Fig 3A). There was a corresponding increase in tumor size for EMT6^siCD200^ tumors growing in WT mice from, ~0.4cm^3^ to ~0.75cm^3^ (Fig 3B), with no significant difference in size of control EMT6 tumors regardless of anti-CD8 treatment. Although the EMT6^siCD200^ tumors were smaller, the % CD45^+^ TILs in PBS-treated WT mice was increased relative to control EMT6 tumors (Fig 3C-see also Fig 2A). However, after treatment with anti-CD8, the level of CD45^+^ TIL fell in both control and EMT6^siCD200^ tumors relative to untreated mice, in parallel with the increase in tumor size of EMT6^siCD200^, again consistent with the notion that CD8^+^ T cells play an important role in anti-tumor immunity-note there was no significant change in size of control EMT6 tumors after anti-CD8 treatment, despite the fall in CD45^+^ TIL.

**Fig 4:**
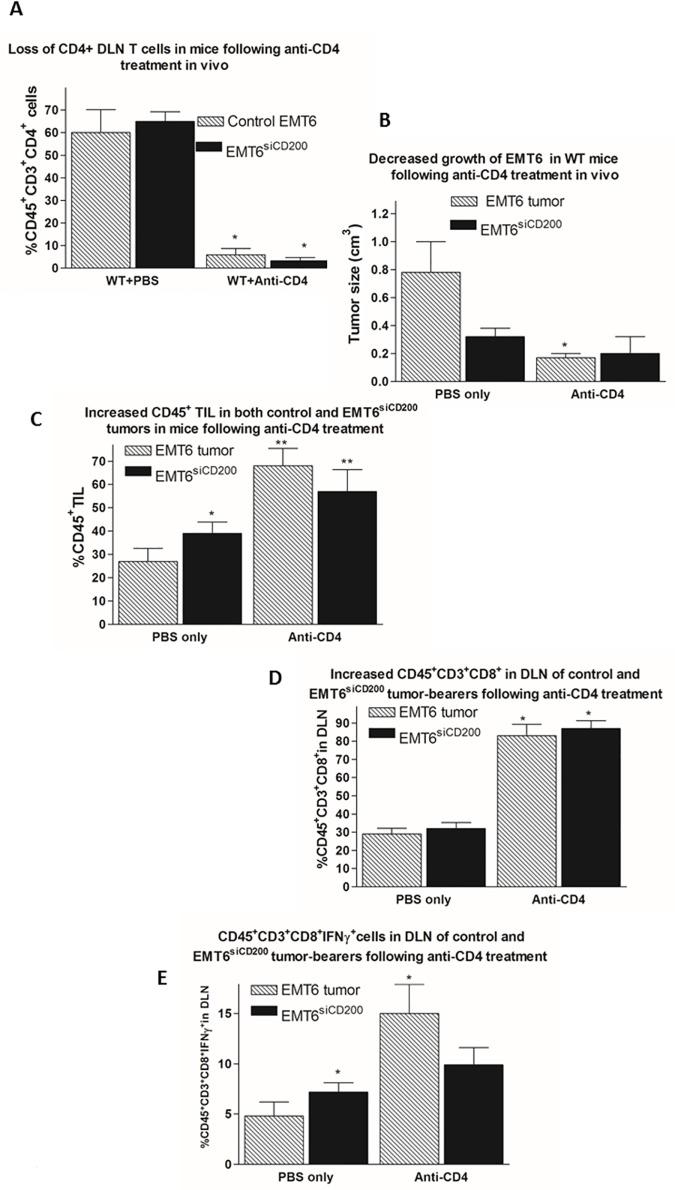
Effect of depletion of CD4^+^ T cells in EMT6 and EMT6^siCD200^ tumor bearing WT mice. All data show represent means (±SD) of 5 mice/group unless otherwise stated. A. Single cell suspensions of DLN from EMT6 and EMT6^siCD200^ tumor bearing mice (day 19 of sacrifice) treated with anti-CD4 or PBS were stained for CD45, CD3, and CD4 cell surface expression. *p<0.05, compared with respective PBS control. B. Tumor size in mice in experiments of the type shown in panel A-data pooled from 2 studies. *p<0.05, compared with respective PBS control. C. EMT6 and EMT6^siCD200^ tumors were harvested from mice as in panel A. Tumors were digested, stained for CD45 cell-surface expression, and analyzed by flow cytometry. *p<0.05, compared with respective with control EMT6 in PBS treated mice; ** p<0.05 compared with respective PBS control. D. Single cell suspensions of DLN from EMT6 and EMT6^siCD200^ WT tumor bearing mice from panel A were stained for CD45, CD3, and CD8 cell surface expression. *p<0.01, compared with respective PBS control. E. DLN cells from EMT6 and EMT6^siCD200^ WT tumor bearing mice from panel D were incubated with GolgiPlug for 8 hours and stained with CD45, CD3, and CD8 cell-surface antibodies as well as an anti-IFNγ intracellular antibody. Data show mean (±SD) of 3 mice/group. *p<0.05 compared with PBS treated mice with control EMT6 tumor.

When CD4^+^ T cells in EMT6 and EMT6^siCD200^ tumor bearing WT hosts were depleted (Fig 4A), a reduction in EMT6 tumor growth was observed with a trend to similarly decreased growth of EMT6^siCD200^ tumors (Fig 4B). A significant increase in the % CD45^+^ TILs in both tumors was observed (Fig 4C). Treatment with anti-CD4 markedly increased the number of CD8^+^ T cells in the DLN of both EMT6 and EMT6^siCD200^ tumor bearing mice (Fig 4D). These data are consistent with the hypothesis that CD4^+^ cells in TIL, possibly Tregs, contribute to tumor growth; that CD4 depletion is associated with augmented numbers of CD8^+^ TILs and decreased tumor growth; and that these effects may be influenced by tumor CD200 expression. Further characterization of CD45^+^CD3^+^CD8^+^ cells in the DLN of tumor bearing mice showed that in PBS-treated EMT6^siCD200^ tumor bearing hosts, CD8^+^ cells expressed more IFNγ than the corresponding cells from control EMT6 tumor bearing hosts (Fig 4E-left hand bars). After treatment with anti-CD4 the number of IFNγ^+^CD8^+^ T cells in the DLN of mice with control EMT6 tumors was increased ~3-fold relative to PBS treated mice, in keeping with the decreased tumor growth (Fig 4B). In contrast, the % IFNγ^+^CD8^+^ cells in mice with EMT6^siCD200^ tumors was not significantly altered (see Fig 4E). Taken together these results support the hypothesis that CD4^+^ T cells play an immunoregulatory tumor-promoting role in mice with EMT6 tumors, while CD8^+^ cells play a protective role in attenuating tumor growth-and that CD200 expression by tumor cells further modulates these effects.

### Metformin augments host resistance to EMT6 tumors in combination with manipulation of the CD200:CD200R axis of immunoregulation

**Fig 5:**
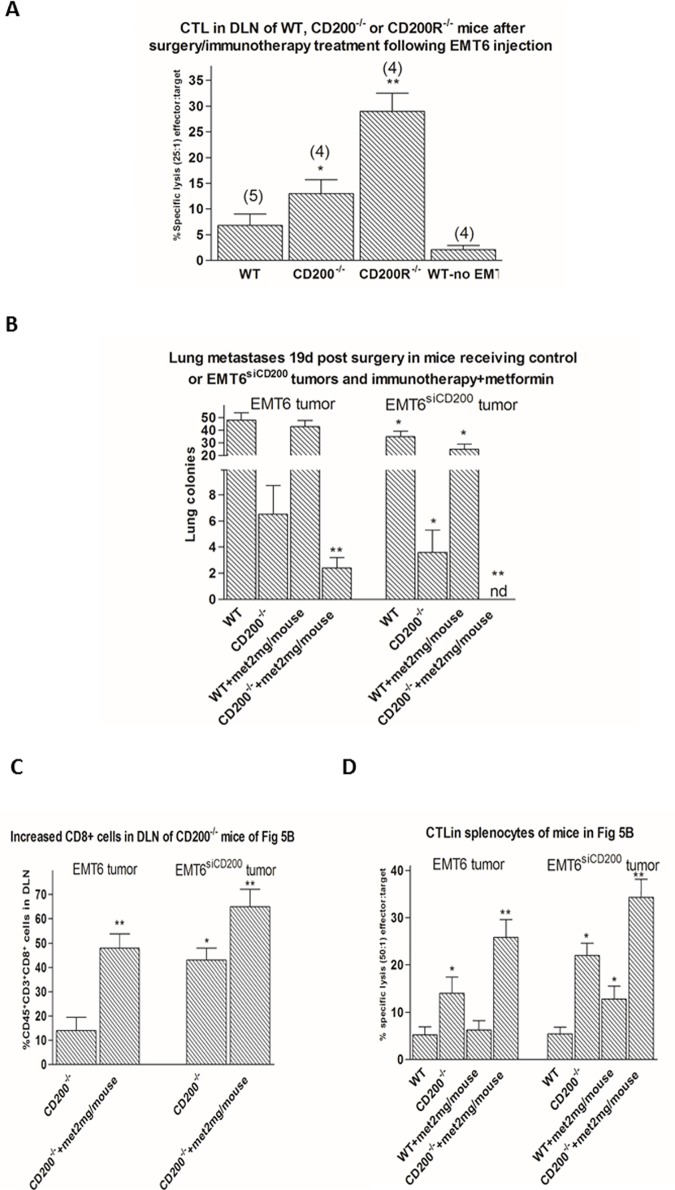
A. WT, CD200^-/-^, and CD200R^-/-^ BALB/c mice received 5x10^5^ EMT6 tumor cells injected into the mammary fat pad. 15 days later tumors were surgically resected and mice were immunized with irradiated EMT6 cells and CpG as adjuvant [4]. Data show specific lysis (^51^Cr release from 2x10^4^ EMT6 target cells at18 hrs) by 5x10^5^ DLN cells, assayed in triplicate from individual mice sacrificed 14d post surgery. All data show arithmetic means ±SD for each group, with the number of mice/group indicated in parentheses. No lysis was seen from any group after treatment of cells (before assay) with anti-CD8 antibody+ complement (data not shown). * p<0.05 compared with WT control; ** p<0.05 compared with CD200^-/-^. B. Decreased lung metastasis in CD200^-/-^ mice receiving EMT6^siCD200^ tumors and undergoing surgery/immunotherapy protocol. This effect was further pronounced by additional metformin treatment after surgery/immunotherapy. Data represent mean±SD for 4 mice/group. *,** p<0.05 compared with control tumor in WT or CD200^-/-^ mice, or corresponding mice without metformin). C. Single cell suspensions of DLN from individual CD200^-/-^ mice with control or EMT6^siCD200^ tumors with/without metformin treatment were stained for CD45, CD3, and CD8 cell surface expression. Data show mean±SD. *, **p<0.05 compared with mice with control tumor or mice not receiving metformin respectively). D. Increased CTL activity in splenocytes of mice shown in Fig 5B. All data are means±SD from assays (in triplicate) using splenocytes from individual mice (4/group). *, **p<0.05 compared with WT mice mice with control tumor or corresponding mice not receiving metformin respectively).

We reported earlier that CD200R^-/-^ mice can be cured of EMT6 tumor, including metastatic tumor growth even up to >300 days post surgery, using combined surgery/immunotherapy. This was attenuated by infusion of both anti-CD4 or anti-CD8 antibody into mice undergoing this "curative" protocol [4], and protection was associated with increased CD8^+^ CTL in DLN of treated mice (Fig 5A), consistent with the correlation in Fig 3 with improved host resistance and increased %CD8^+^ cells in DLN in mice with EMT6^siCD200^ tumors. In contrast to these data, incomplete tumor resistance was seen after surgery/immunotherapy in CD200^-/-^ mice, with metastatic growth in lungs and liver detected by 150 days post surgery [4]. We attributed this incomplete protection seen in CD200^-/-^ mice to an ongoing immunosuppression by tumor rather than host-expressed CD200 [4]-see also decreased CTL in CD200^-/-^ (compared with CD200R^-/-^) mice in Fig 5A. The improved resistance seen following silencing of tumor CD200 expression is well documented in Fig 1, using EMT6^siCD200^ tumors in WT mice. While CD200^-/-^ mice were cured of tumor growth using conventional chemotherapy with a combination of paclitaxel and anti-VEGF antibody, this treatment was not affected by anti-CD4 or anti-CD8 treatment, and did not lead to development of any immunity to re-challenge with the same tumor [5].

In the following studies we explored whether immunotherapy and chemotherapy could be manipulated to enhance tumor resistance, by modulating host and/or tumor CD200 expression, and using metformin as a novel chemotherapeutic. As noted earlier, metformin, a non-conventional anticancer agent, has been reported to improve the outcome in a number of human cancers when administered together with standard chemotherapy [13].

Control EMT6 or EMT6^siCD200^ tumors in WT or CD200^-/-^ mice were resected 15 days after tumor cell injection. Two days later, mice were immunized with 2x10^6^ irradiated EMT6 cells and CpG as adjuvant, and treated with i.p. metformin injections daily (2mg/mouse). The combination of metformin with immunotherapy, and lack of CD200 expression in either tumor (EMT6^siCD200^ tumors in WT mice) or host (CD200^-/-^ mice) resulted in increased survival and prevention of lung metastasis compared with either immunotherapy or metformin treatment alone. Optimal responses were seen using EMT6^siCD200^ tumors injected into CD200^-/-^ mice (Fig 5B) where no lung metastases were detected. When we examined CD8^+^ cells in DLN of tumor-bearing CD200^-/-^ mice (Fig 5C), we found that metformin increased the numbers of CD8^+^ cells in DLN regardless of growth of control EMT6 or EMT6^siCD200^ tumors. No such changes were observed in WT mice (data not shown). Moreover, in 18-hr ^51^Cr release cytotoxicity assays using splenocytes from mice shown in Fig 5B, we found metformin increased the cytotoxicity measured even in WT mice when EMT6^siCD200^ tumors were implanted, while in CD200^-/-^ mice (which in all cases showed greater cytotoxicity than corresponding groups of WT mice), metformin augmented cytotoxicity regardless of growth of control EMT6 or EMT6^siCD200^ tumors (Fig 5D). These data imply that immunotherapy produces its greatest effect in mice in which disruption of a CD200:CD200R suppressive signal is impeded (using CD200^-/-^ hosts, and/or EMT6^siCD200^ tumors) and when additional chemotherapy is given using metformin-both factors augment numbers and functional activity in CD8^+^ cells, with CD200^-/-^ mice also expressing less CD4+Tregs in TILs.

### Autophagy may represent a key mechanism for metformin-associated enhanced tumor host resistance in CD200^-/-^ mice receiving EMT6 tumors

**Fig 6:**
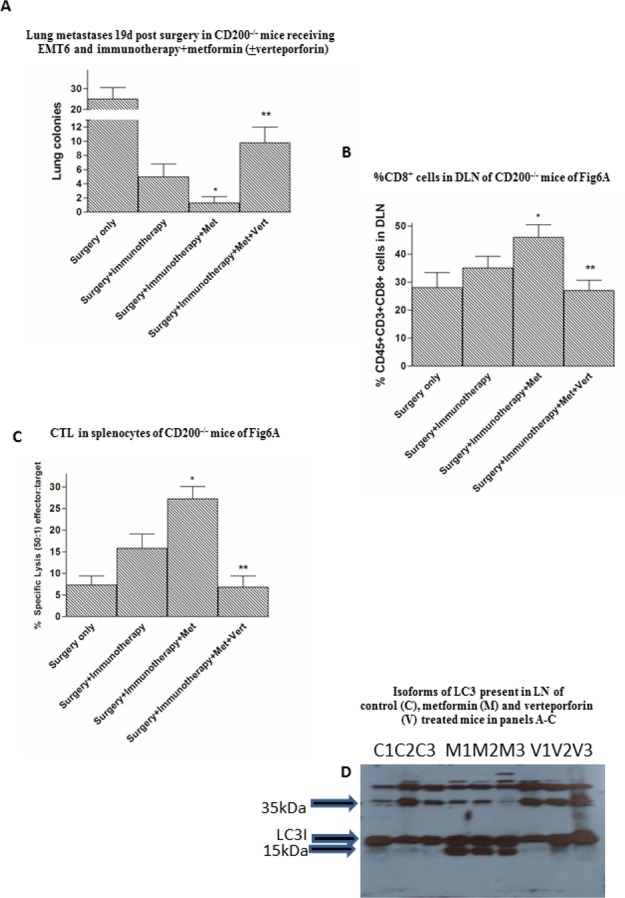
Effect of verteporfin on metformin-associated augmentation of host resistance to EMT6 tumor growth and metastasis in CD200-/- mice. A. CD200^-/-^ EMT6 tumor bearing mice immunized with irradiated EMT6 cells and CpG as adjuvant after primary tumor resection, with and without daily administration of metformin and verteporfin (an autophagy inhibitor), were sacrificed 19d after surgery and tumor nodules in lung evaluated macroscopically after fixation in Bouin’s solution. B. Single cell suspensions of DLN from mice in panel A were stained for CD45, CD3, and CD8 cell surface expression. C. CTL activity against EMT6 tumor cells in 18hr ^51^Cr release assays using splenocytes of CD200^-/-^ mice (50:1 effector:target) shown in panels A and B. In all panels data show mean (±SD) of 4 mice/group. *p<0.05 compared with group receiving surgery and immunotherapy only (no metformin).**p<0.005 compared with group receiving surgery, immunotherapy and metformin. D. Western blots showing detection of LC3-I (16–18 kDa–top band) and LC3-II (14–16 kDa–bottom band). in LN taken from individual control (C), metformin-treated (M) or metformin and verteporfin-treated (V) mice shown in previous panels. Anti-LC3 (1:200); anti-rabbit HRP (1:5000); 10 minute exposure.

Recent studies focusing on the possible mechanism(s) of action of metformin in cancer therapy have suggested that its role in blockade of apoptosis and enhancement of autophagy may be of importance [14]. Metformin treatment was reflected in increased CD8^+^ TILs with decreased exhaustion and apoptosis. Accordingly, in a final series of studies we treated CD200^-/-^ mice with EMT6 tumors, receiving surgery and immunotherapy as before, with either metformin alone (see Fig 5B) or metformin along with an autophagy blocking agent, verteporfin (the latter was given at 2d intervals with metformin). Control CD200^-/-^ mice received only surgical resection of tumor. Sacrifice of all mice 19d after surgical removal of the primary tumor showed that verteporfin blocked the effect of metformin in attenuating EMT6 metastasis to lungs in CD200^-/-^ mice (Fig 6A), and decreased both the %CD8^+^ cells in DLN (Fig 6B) and CTL activity in splenocytes (Fig 6C) to baseline levels seen in CD200^-/-^ mice receiving only surgery and immunotherapy. Moreover, Western blots of protein extracted from lymph nodes of these same mice, and probed for isoforms of LC3, a technique widely used to monitor autophagy, revealed that the increased LC3 conversion (LC3-I to LC3-II) seen in the presence of metformin was attenuated by verteporfin in these mice (Fig 6D). These data are consistent with the notion that an important mechanism of action of metformin involves enhancement of auto phagocytosis, which in turn synergizes with immunotherapy to produce an effective anti-CD8^+^ tumor immune response.

## Discussion

CD200:CD200R1 interaction has been shown to suppress immune responses in autoimmune disorders, infectious diseases, transplantation, and cancer. CD200^-/-^ mice were first generated on a C57BL/6 (B6) background in 2000 [15]. These mice had an increased susceptibility to autoimmune diseases, including EAE and CIA, consistent with the hypothesis of a general immunoregulatory role for CD200. CD200^-/-^ B6 mice were also found to be more susceptible to viral infections, such as influenza, where a dose causing non-fatal disease in WT mice now resulted in death [16]. In the studies described herein, we used our own CD200^-/-^ BALB/c mice and WT BALB/c mice as EMT6 and EMT6^siCD200^ tumor bearing hosts to investigate how the lack of CD200 expression by host and tumor cells affected EMT6 breast cancer progression.

EMT6 tumor growth in CD200^-/-^ hosts did not differ significantly from WT mice, indicating that the lack of CD200 expression in the host did not significantly perturb host resistance, unlike lack of the receptor, CD200R, where CD200R^-/-^ mice were found to show increased resistance [3]. Lack of CD200 expression by EMT6 tumor cells, in contrast, resulted in reduced cancer progression in both WT and CD200^-/-^ mice (Fig 1)-see also [3]. These data imply that CD200 expression by tumor cells themselves is more important than host CD200 expression for control of local tumor growth in this breast cancer model using CD200R^+^ recipients.

More detailed analysis of tumor growth in these mice showed that while EMT6^siCD200^ tumors were smaller than control EMT6 tumors, they had a greater percentage of CD45^+^ infiltrating immune cells (Fig 2A), suggesting an enhanced immune response in hosts bearing tumors with reduced CD200 expression. CD45^+^ cells infiltrating the EMT6^siCD200^ tumors (TILs) were enriched in myeloid cells (Fig 2B) and in CD8^+^cells (Fig 2C). In contrast, EMT6^siCD200^ tumor bearing mice had lower numbers of CD8^+^ T cells in the DLN, implying an increased trafficking to the periphery and tumors (Fig 2D), with a notable decrease in cells with Treg phenotype also evident in DLN (Fig 2E). Interestingly, the EMT6 tumor microenvironment contained more inflammatory cytokines, including IL-1β and GM-CSF, than EMT6^siCD200^ tumors (Fig 2F), which is consistent with a role for these cytokines in promoting tumor growth in the EMT6 breast cancer model.

GM-CSF is secreted by a variety of cells, including macrophages, granulocytes, T cells, endothelial cells, and fibroblasts, and is known to have pleiotropic effects. This cytokine has been reported to have tumor promoting and protective roles in breast cancer progression under different conditions [17,18]. GM-CSF has been shown to be capable of stimulating an immune response, improving the antigen presenting capacity of DCs, as well as of suppressing an immune response by favoring the development of immature DCs that recruit CD4^+^CD25^+^FOXP3^+^ T_reg_ [18]-see Fig 2E. A recent study in breast cancer patients reported an association of elevated GM-CSF levels and EMT and poor prognosis [19]. IL-1β is an inflammation-associated cytokine secreted mainly by macrophages localized in the breast tumor microenvironment [20], whose overexpression is thought to be tumor-promoting in many cancer models [21]. In murine models of breast cancer it has been shown that IL-1β is involved in various stages of tumor development, invasiveness, and metastasis [22, 23], with one mechanism involving augmentation of MDSC expansion [23]-see Fig 2B.

Further analysis of the role of T cells in host tumor resistance came from analysis of the progression of EMT6 and EMT6^siCD200^ tumors in WT mice treated with CD4 and CD8 depleting antibodies 6 days after tumor cell injection. Depletion of CD8^+^ cells caused an increase in tumor size (Fig 3B) and a decrease in immune cell infiltration (Fig 3C), while in contrast depletion of CD4^+^ T cells reduced growth of both EMT6 and EMT6^siCD200^ tumors (Fig 4B), along with an increase in CD45^+^ TILs (Fig 4C) and increased CD8^+^ cells in DLN (Fig 4D), suggesting that the depleted CD4^+^ cells may exert an immunosuppressive effect in tumor bearers. Increased numbers of IFN-γ expressing CD8^+^ T cells were seen in the DLN of EMT6 though not EMT6^siCD200^ tumor-bearing mice following treatment with anti-CD4, supporting a role for such cells in host resistance (Fig 4E).

We previously showed that female CD200R^-/-^ BALB/c mice could be cured from EMT6 breast cancer by immunization with irradiated EMT6 cells and CpG after surgical resection of the primary tumor [4], with no metastases (macroscopic lung nodules or microscopic nodules detected by limiting dilution analysis of DLN) apparent >300days post surgery. In contrast WT mice treated under the same protocol died within 20-30d post surgery, while CD200^-/-^ mice also failed to survive beyond 100-150d. Interestingly, the induced immunity seen in CD200R^-/-^ mice was dependent upon both CD4^+^ and CD8^+^ cells, with many parallels to the data reported above [4]. Indeed, a marked increase in cytotoxic T cells was found in DLN of CD200^-/-^ and CD200R^-/-^ mice post-surgery/immunotherapy, compared with similar DLN of WT mice, or of WT mice receiving no surgery/immunotherapy (Fig 5A).

While conventional chemotherapy including paclitaxel and anti-VEGF cured primary EMT6 tumors in WT mice, no effective anti-EMT6 immunity was demonstrated in such mice [5], or in similarly treated CD200^-/-^ or CD200R^-/-^ mice-unpublished. This led us to investigate the possibility of using non cytotoxic drugs as adjunctive therapy in mice receiving immunotherapy post surgical de-bulking of tumor. Numerous observational studies have reported decreased cancer incidence and cancer-related mortality in diabetics receiving standard doses of metformin (1500 to 2250 mg/day in adults [24, 25]. An epidemiological study of 2,529 women with breast cancer reported higher pathologic complete response rates (considered a surrogate for overall survival in this setting) to neoadjuvant systemic therapy in diabetic patients receiving metformin compared to diabetic patients not receiving metformin and non-diabetic patients not receiving metformin [26]-no improvement in the estimated 3-year relapse-free survival rate has been reported for breast cancer patients on metformin.

Studies reported above (Fig 5B) show that there is indeed an additive protective effect on long term anti-tumor immunity seen in CD200^-/-^ mice following combination of the immunotherapeutic approach used before, with daily metformin administration beginning following immunotherapy. Thus CD200^-/-^, but not WT, mice showed a significant decrease in lung metastasis after initial challenge with control EMT6 (left hand panel Fig 5B), while after challenge with EMT6^siCD200^ tumors no lung metastases were detected at the time of sacrifice (right hand panels Fig 5B). These latter changes occurred in parallel with increased numbers of CD8^+^ cells in DLN (Fig 5C), and a marked increase in CTL activity in splenocytes of mice receiving both immunotherapy and metformin even in WT mice receiving EMT6^siCD200^ tumors (Fig 5D). It should be noted that in the absence of additional immunotherapy as used in these studies, use of metformin alone in either WT or CD200^-/-^ mice receiving EMT6 tumors did not lead to any significant attenuation of tumor growth (RMG-unpublished).

One possible mechanism of CD8^+^ T cell immune response augmentation is through induction of autophagy mediated by metformin. Multiple studies report that metformin activates AMPK, inhibits mTOR, and promotes autophagy. Autophagy is best described as a generalized degradation of cytoplasmic components. Double-membrane vesicles, called auto phagosomes, then transport unwanted cell components to the lysosomes in an inner autophagic membrane. These vesicles fuse to liberate the autophagic body and its contents into the lumen of the vacuole for degradation. While multiple proteins (>16 so far detected) have been implicated in autophagy, LC3 forms a stable association with the membrane of auto phagosomes. LC3 exists as two forms, a cytoplasmic form LC3-I, and a membrane-bound form LC3-II, which is converted from LC3-I, and is believed to initiate formation and lengthening of the auto phagosome. Enhanced autophagy has been reported to be essential for CD8^+^ T cell memory cell survival [27], and this could explain the significant increase in the number of CD8^+^ cells in the DLN of CD200^-/-^ mice treated with metformin after tumor resection and immunization. This notion was in turn supported by data from an experiment using verteporfin, a drug that inhibits early stages of autophagy without light activation [28] (Fig 6). Western blots of LN taken from these mice showed that detection of the LC3 II isoform was increased in mice treated with metformin, an effect attenuated by verteporfin (Fig 6D), and these effects paralleled the augmented tumor protection, and CD8^+^ cytotoxicity in metformin treated mice (Fig 6A–6C), again effects abolished by verteporfin.

In summary, our data support the hypothesis that CD200 blockade may be an effective therapeutic target in breast cancer. Currently, the majority of immunotherapies in clinical use are adjuvant treatments to chemotherapy or radiation therapy, but there are over 1000 ongoing clinical trials of cancer immunotherapies [29], There is a strong rationale to continue to develop combinatorial strategies targeting mechanisms of immunosuppression that synergize with standard treatments as well as unconventional drugs, like metformin, to improve the outcome for cancer patients.

## Supporting information

S1 Fig Phenotypic analysis of CD200^-/-^ mice. A) Levels of sCD200 in the peripheral blood of WT mice and CD200^-/-^ mice as assessed by ELISA. Data show mean (**±**SD) of 3 mice/group. B) Flow cytometric analysis of cell-surface expression of CD200 by cells in spleen, thymus, and lymph nodes in WT (n = 3) and CD200^-/-^ (n = 6) mice. Shaded curves show tissue staining from CD200^-/-^ mice. Representative plots are shown. As reported for CD200^-/-^ BL/6 mic^e^, the frequency of immune cells in CD200^-/-^ BALB/c mice, including B cells, CD4^+^ and CD8^+^ T cells, NK cells, macrophages, and myeloid cells, did not differ significantly from WT BALB/c mice. No abnormalities were detected in reproductive cycles and the health of litters from CD200^-/-^ female BALB/c mice.
(TIF)
