# Supplementary figures and images for: Importance of CD200 expression by tumor or host cells to regulation of immunotherapy in a mouse breast cancer model
Source: PLoS One. 2017 Feb 24;12(2):e0171586. doi: 10.1371/journal.pone.0171586 (PMC5325206; doi:10.1371/journal.pone.0171586)

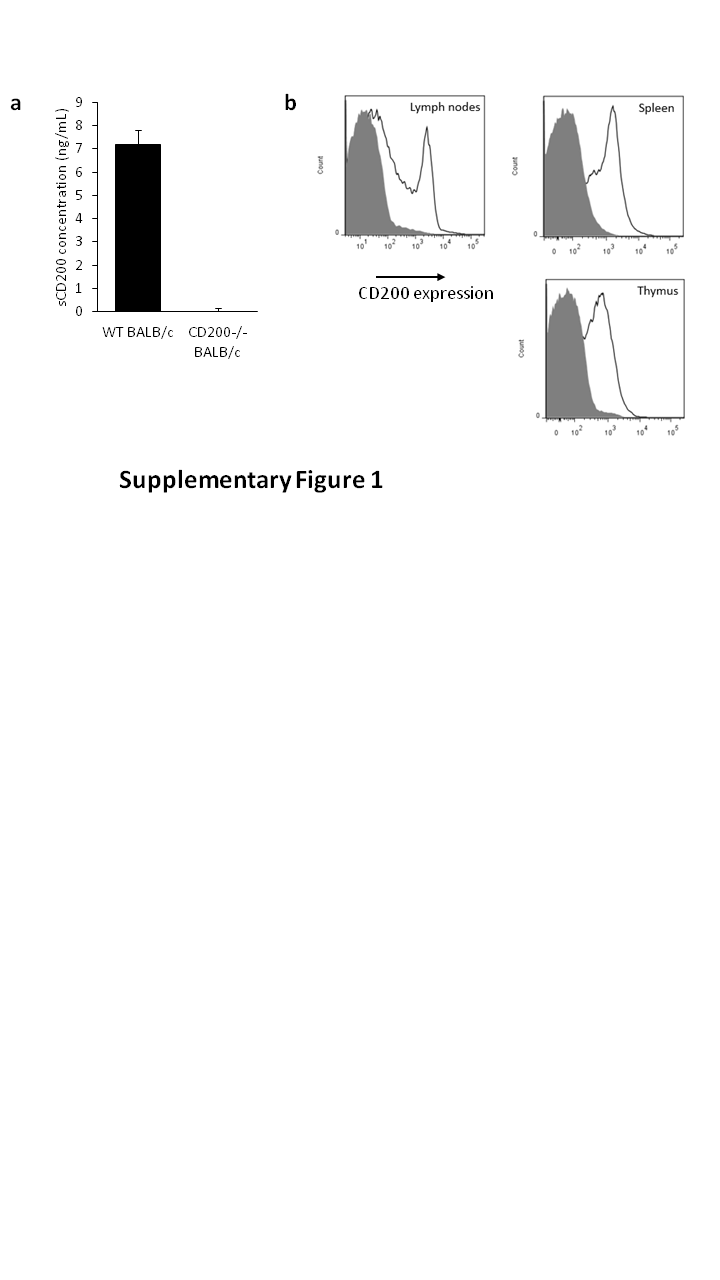

Supplement: S1 Fig — A) Levels of sCD200 in the peripheral blood of WT mice and CD200-/- mice as assessed by ELISA. Data show mean (±SD) of 3 mice/group. B) Flow cytometric analysis of cell-surface expression of CD200 by cells in spleen, thymus, and lymph nodes in WT (n = 3) and CD200-/- (n = 6) mice. Shaded curves show tissue staining from CD200-/- mice. Representative plots are shown. As reported for CD200-/- BL/6 mice, the frequency of immune cells in CD200-/- BALB/c mice, including B cells, CD4+ and CD8+ T cells, NK cells, macrophages, and myeloid cells, did not differ significantly from WT BALB/c mice. No abnormalities were detected in reproductive cycles and the health of litters from CD200-/- female BALB/c mice. (TIF) [file pone.0171586.s001.tif]
